# MEDICARE-MEDICAID DUAL ELIGIBLE SPECIAL NEEDS PLANS (D-SNPS): ENROLLMENT TRENDS (2007–2023)

**DOI:** 10.1093/geroni/igae098.2462

**Published:** 2024-12-31

**Authors:** Roshani Dahal

**Affiliations:** University of Minnesota School of Public Health, Minneapolis, Minnesota, United States

## Abstract

Dual-Eligible Special Needs Plans (D-SNPs), private Medicare Advantage plans, exclusively enroll Medicare-Medicaid dual enrollees. First operationalized in 2006 and popular among state Medicaid agencies, enrollment in integrated plans remains a challenge. CMS expects states to transition existing plans into D-SNPs by end of 2025. We used publicly available SNP monthly data (2007-2023) to examine enrollment growth. CMS began reporting on integration status (2022) of D-SNPs: Coordination-Only, Highly-Integrated (HIDE), and Fully-Integrated (FIDE). D-SNPs began with 0.67million duals (73.9% of SNP enrollment). A moratorium on SNPs (2008-2009) correlates with slight decline; however, Affordable Care Act (2010) lifted moratorium and legislation extended SNPs through 2018, resulting in higher enrollment (mean 81.7%). In February 2018, SNPs were permanently authorized and annual enrollment in D-SNPs has consistently increased (11% to 16%). As of December 2023, there were 5.69 million duals (+16.4%) in D-SNPs, representing 89.5% of SNP enrollment. Of 823 plans, 60.6% were Coordination-Only, 29.8% were HIDEs, and 8% were FIDEs. Only twelve states offered FIDE-SNPs. Percentage of integrated plans have declined in the last year, despite overall increase in all D-SNPs (Coordination-Only plans grew by 34%, FIDE 15.7%, HIDE 12.5%). D-SNP market has experienced consistent high growth rate, outpacing growth in Medicare Advantage (8%). Although designed to serve duals, focused on integrating Medicare-Medicaid benefits, less than 10% D-SNPs are fully integrated more than 15 years later. Although HIDE-SNPs (a higher level of integration) began only in 2021, almost 30% of duals are enrolled. Integration under D-SNPs is important for reducing fragmented care among duals.

